# BRCA-Associated Ovarian Cancer: From Molecular Genetics to Risk Management

**DOI:** 10.1155/2014/787143

**Published:** 2014-07-22

**Authors:** Giulia Girolimetti, Anna Myriam Perrone, Donatella Santini, Elena Barbieri, Flora Guerra, Simona Ferrari, Claudio Zamagni, Pierandrea De Iaco, Giuseppe Gasparre, Daniela Turchetti

**Affiliations:** ^1^Dipartimento di Scienze Mediche e Chirurgiche, Unità di Genetica Medica, Università di Bologna, Policlinico S. Orsola-Malpighi, Via Massarenti 9 [Pad. 11, 2° Piano], 40138 Bologna, Italy; ^2^SSD Oncologia Ginecologica, AOU di Bologna, Policlinico S. Orsola-Malpighi, 40138 Bologna, Italy; ^3^UO Anatomia Patologica, AOU di Bologna, Policlinico S. Orsola-Malpighi, 40138 Bologna, Italy; ^4^SSD Oncologia Medica, AOU di Bologna, Policlinico S. Orsola-Malpighi, 40138 Bologna, Italy; ^5^Dipartimento Di Scienze e Tecnologie Biologiche ed Ambientali, Università del Salento, 73100 Lecce, Italy; ^6^UO Genetica Medica, AOU di Bologna, Policlinico S. Orsola-Malpighi, 40138 Bologna, Italy

## Abstract

Ovarian cancer (OC) mostly arises sporadically, but a fraction of cases are associated with mutations in BRCA1 and BRCA2 genes. The presence of a BRCA mutation in OC patients has been suggested as a prognostic and predictive factor. In addition, the identification of asymptomatic carriers of such mutations offers an unprecedented opportunity for OC prevention. This review is aimed at exploring the current knowledge on epidemiological and molecular aspects of BRCA-associated OC predisposition, on pathology and clinical behavior of OC occurring in BRCA mutation carriers, and on the available options for managing asymptomatic carriers.

## 1. The BRCA1 and BRCA2 Genes

### 1.1. Functions and Structure

BRCA1 and BRCA2 are separate genes mapping on two different chromosomes (17q21 and 13q12.3, resp.). They have distinctive primary sequences nevertheless disruption of either BRCA gene leads to similar pathophysiological effects [1], as well as to similar cancer spectra.

BRCA1 and BRCA2 are considered tumor suppressor genes, since they are deputed to the maintenance of genomic stability and hence to the control of cell growth [2]. The BRCA1 and BRCA2 proteins are mainly involved in the repair of DNA double-strand breaks (DSBs) via the homologous recombination (HR) pathway [3, 4]. DSBs are repaired by two major pathways: nonhomologous end-joining (NHEJ) and HR [4]. NHEJ usually results in changes in the DNA sequence at the break site [4]. In presence of a double-strand break, HR allows the exchange of the same genetic sequence from the healthy homologous sister chromatid to the damaged one [5] and therefore generally results in accurate repair of the break [6]. Both BRCA1 and BRCA2 proteins are critical for the recovery of DSBs by HR. Deficiency of BRCA1 or BRCA2 function leads to a high degree of chromosome instability, such as chromosome breaks, severe aneuploidy, and centrosome amplification [7–9] probably because it triggers the use of alternative pathways for the repair of DSBs such as NHEJ, resulting in accumulation of mutation events [4]. Genetic aberrations occur spontaneously favored by DNA-damaging agents that induce DSBs, in particular DNA cross-linking agent, mitomycin or platinum compounds [10], which explains why OC patients carrying BRCA1 or BRCA2 mutations display a better response to platinum-based chemotherapy when compared to patients with sporadic OC [11, 12].

Several proteic interactors of BRCA1 and BRCA2 have been identified. RAD51 is responsible for repair mechanism of DSBs and is one of the most important players in HR; its functions are ultimately complemented by the proteins encoded by the two BRCA genes [13, 14]. A number of studies demonstrated the BRCA2 role in the regulation of intracellular transport, enzymatic activity, and function of RAD51 [15]. BRCA1 exhibits a physical association with RAD51 to create a complex responsible for resected single-stranded DNA at double-strand repair sites [16]. Other studies suggest a BRCA1 role in altering chromatin structure in the presence of a DNA damage to allow access for repair. It was shown that following damage, histone H2AX becomes extensively phosphorylated and forms foci at break sites [17]. BRCA1 is recruited to these foci before every other factor, such as RAD51, suggesting that H2AX and BRCA1 initiate repair by modifying local chromatin structure, thereby allowing DNA repair proteins to access the damage site [18]. Moreover, BRCA1 and BRCA2 exhibit a transcriptional coregulator and chromatin remodeling function [14, 19] and BRCA1 seems to have the ability to coactivate endogenous p53-dependent stimulation of p21 [20].

BRCA1 is a very large gene that generates several different transcripts. The full-length form is a 2843 amino acids (p220) protein and a shorter (1399 amino acids) form, named BRCA1-IRIS, may have an oncogenic activity. BRCA2 is even larger, counting 3418 amino acids, but has fewer recognized motifs [21]. BRCA1 and BRCA2 genomic regions harbor a very high density of repetitive DNA elements that contribute to genetic instability [22]. In particular the BRCA1 region consists of 42% Alu sequences and 5% non-Alu repeats [23]. The BRCA2 genomic region is 47% repetitive DNA: 20% Alu sequences and 27% LINE and MER repetitive DNA. Alu-dense regions of the genome are associated with a high density of genes and localize predominantly to R bands of metaphase chromosomes, which are involved in homologous and nonhomologous chromosomal exchange [24]. Based on the density of repeat elements in these genes, Alu-mediated genomic rearrangements within BRCA1 and genomic rearrangements in BRCA2 have been observed [25, 26].

### 1.2. Mutational Analysis

Disease-associated mutations are scattered across the entire length of the BRCA1 and BRCA2 genes and usually result in a truncated protein. Deleterious missense mutations occur frequently in exons-encoding domains that interact with BRCA1-binding proteins, such as BARD1, BRIP1, and PALB2, which (along with RAD51C, RAD51D, and possibly RAP80 and FAM175A) are also breast and/or ovarian cancer susceptibility genes [21].

The BRCA genes are routinely tested by Sanger sequencing of exons and exon-intron junctions. Whether a clearly pathogenic variant has not been identified, the multiplex ligation probe assay (MLPA) should be applied in order to exclude the presence of large BRCA1 deletions, involving one or more full-length exons. The MLPA assay is a rapid and robust method for copy number quantification and methylation status analysis of genomic sequence. It can be easily multiplexed and requires only a small amount of input DNA [26].

The silencing of BRCA1 gene trough promoter hypermethylation may occur in sporadic breast and ovarian cancers [27]. The analysis of DNA methylation patterns of BRCA1 may also be a useful predictive marker of the response to the PARP1 inhibitor therapy [28].

In a few years the next generation sequencing (NGS) technology, including different high-throughput sequencing systems, will likely replace Sanger sequencing as the technique of choice for genetic testing of BRCA genes because it undoubtedly offers advantages in terms of sensitivity, scale, and costs. However, the large number of false positive/negative insertions and deletions (indels) due to the high frequency of homopolymers in BRCA genes, which cause sequencing errors, has slowed down the usage of NGS for clinical genetic testing. The specificity of indels detection in NGS data is improving by the application of different filtering criteria for the variants calling [29].

Based on the diagnostic criteria used in the United States to select patients to be tested, clearly pathogenic mutations in either BRCA1 or BRCA2 are found in 10% to 15% of hereditary breast and ovarian cancer families. Rare patients with mutations in both BRCA1 and BRCA2 genes were described [30], which has led to recommending that the analysis of both genes should be completed even after the finding of one mutation.

The major challenge in the diagnostic testing of BRCA genes concerns the interpretation of unknown variants, the so-called “variants of uncertain significance” (VUS). The pathogenic variants can be nonsense mutations, small indels causing a frameshift, splicing site mutations that occur inside of the canonical splice sites, large deletions or known deleterious missense variants. VUS are alterations in the DNA sequence that have unknown effects on the protein function and disease risk; they usually are missense substitutions, splicing site mutations that occur outside of the canonical splice sites, small in-frame indels. Their frequency varies depending on the patient's ethnicity: in the United States, in individuals of European ancestry VUS account for approximately 5% of the alterations reported from BRCA genetic testing, but the estimate is as high as 20% among individuals of African ancestry [31]. To guide the clinical management, a statistically rigorous model that provides pathogenicity score for each variant has been proposed by the International Agency on Cancer Research (IARC) of the World Health Organization. It is a five-level system where classes 1 and 2 are managed as neutral variants and classes 4 and 5 are managed as pathogenic variants. Class 3 variants still have insufficient evidence to be considered either neutral or pathogenic and they require reclassification.

The method for VUS classification begins with a prior probability based on an* in silico* evaluation of the effect of each variant at the protein or mRNA level. Observational data, such as personal and family history, cosegregation of VUS with cancer phenotype in pedigrees, cooccurrence with other known pathogenic mutations, tumor immunohistochemistry, and histological grade, are summarized as likelihood ratios in favor of pathogenicity and used to update the prior probability [32, 33]. The VUS database was built on a modified Leiden Open source Variation Database (LOVD) v.2.0 system, providing a flexible environment for the creation of locus-specific databases [34].

## 2. Clinicopathological Features of BRCA-Associated Ovarian Cancer

### 2.1. Prevalence of BRCA1/2 Mutations among Ovarian Cancer Patients

OC patients with a family history of breast or ovarian cancer present high probability of carrying a mutation in BRCA1 or BRCA2. The rate of mutations found in families with multiple cases of breast and ovarian cancer varies from 9% to 46%, depending on selection criteria and ethnicity [35–41]. Among families with at least two cases of epithelial OC, 43% were found to harbor BRCA germline mutations (36% BRCA1 and 7% BRCA2 mutations) [42].

In addition, many efforts have focused on assessing the contribution of BRCA mutation to the total burden of OC. Early population-based studies reported BRCA1 mutation rates in OC patients varying from 1.9 to 7.2% [43–46].

In a recent population-based cohort of 1001 Australian OC patients, 141 (14.1%) were found to carry a pathogenic mutation in BRCA1 (88 patients) or BRCA2 (53 patients), the proportion being 16.6% when only serous carcinomas where considered and increasing to 17.1% in patients diagnosed with high-grade serous cancers. 44% of mutation carriers failed to report any family history of breast or ovarian cancer [47]. Another large population-based study was conducted in Ontario, Canada: among 1342 unselected OC patients, 176 (13%) carried a BRCA1/2 mutation. Higher prevalence of mutations was associated with Italian, Jewish, or Indo-Pakistani origin, serous histology, younger age of onset, and, obviously, a family history of breast or ovarian cancer; among women without family history of such cancers, prevalence was 7.9% [48].

Several smaller studies have been carried out in different countries: in a Greek cohort of 592 patients with sporadic OC, a targeted screening of the commonest BRCA1 mutations detected 27 mutation carriers (4.6%) [49]. In Belgium, de Leeneer et al. tested 193 sporadic cases of breast and ovarian cancer for BRCA1/2, finding 3 carriers among 7 women with both breast and ovarian cancer (42.9%) but none among 6 patients with OC only [50]. In Poland, BRCA1/2 mutations were identified in 21 out of 151 consecutive OC patients (13.9%) [51], while in a sample of 74 Russian patients, the prevalence was as high as 19% [52]. In Korean OC patients, BRCA1/2 mutations were detected in 13 of 40 (33%) reporting a strong family history and in 23 of 283 (8%) without significant family history [53].

### 2.2. Pathology of BRCA-Associated Ovarian Cancer

The vast majority of OC associated with germline BRCA mutations reported in the literature are high-grade and advanced-stage serous carcinomas [54]. Most studies, however, do not include a complete pathology review of all material and rely only on pathology report. Nevertheless, the studies that incorporate a systematic histopathologic review confirm and emphasize the greater frequency of high-grade serous carcinomas in BRCA1-associated tumors, with a frequency ranging from 67% to 100%. Frequencies of endometrioid and clear cell carcinomas seem to be similar to the general population. Although other tumor types have been observed, they are extremely rare, accounting for <10% of all tumors [55–59]. However, these data may have been biased by the most recent updates about pathogenesis and clinicopathologic diagnosis of OC and by the significant interobserver variation that affects histopathological typing of all tumors, including OC, with categorization being particularly difficult for high grade lesions.

Also borderline BRCA1 carriers ovarian tumors are very rare [46], which reinforces the increasing evidence that BRCA1 mutations do not play a role in the development of this type of tumors. Fallopian tube cancer and peritoneal carcinomas are also part of the BRCA-associated disease spectrum.

A large dataset, aimed at expanding the knowledge of OC pathology in these patients, has been recently reported by the Consortium of Investigators of Modifiers of BRCA1/2 (CIMBA) that represent the largest collaborative study of BRCA1 and BRCA2 mutation carriers, involving more than 37 groups from more than 20 countries [60]. Tumor pathology data has been collected through several mechanisms, including medical records and pathology reports. Laboratory methods for tissue preparation, immunohistochemistry and biochemical assays, scoring systems, and data interpretation vary widely; nevertheless data collated by CIMBA seem to be more representative of typical assessment of pathology conducted in routine practice. CIMBA results confirm that over 70% of OCs in BRCA1 and BRCA2 mutation carriers are grade 3 serous carcinoma (Table 1).

Moreover, according to the recent update on the different pathogenesis and clinicopathologic features of ovarian low-grade and high-grade serous carcinomas, as well as to the fundamental molecular differences between both categories of tumors, the vast majority of BRCA-related hereditary ovarian tumors are high-grade serous carcinoma. Low-grade serous carcinoma and noninvasive micropapillary serous carcinoma do not seem to be related to germline mutations of BRCA [61].

### 2.3. Molecular Pathology of BRCA-Associated Ovarian Cancer

A high frequency of loss of heterozygosity (LOH) in or near the BRCA1 region has been reported in BRCA1-mutation positive OC [62]. In addition, LOH of BRCA, as well as TP53 mutations, have been demonstrated as early events in high-grade serous carcinomas in patients with germline mutations; accordingly, mutations and/or loss of heterozygosity of TP53 and BRCA have been identified in early carcinomas and epithelial inclusions of the ovary [63, 64]. More recently, attention has been drawn to a lesion in the fallopian tube that has the cytologic appearance of high-grade serous carcinoma of the ovary and has been designated tubal intraepithelial carcinoma (TIC). These lesions are almost always detected in the fimbriae of the fallopian tube. The fimbriae are in close proximity to the ovarian surface, and it has been suggested that the tube is the origin of a subset of “ovarian” high-grade serous carcinomas. This is supported by some data: early serous carcinomas in prophylactic bilateral salpingooophorectomy specimens from women with BRCA mutations have been detected in the tube, especially the fimbriae, in the absence of an ovarian tumor; identical TP53 mutations have been reported in TIC and synchronous ovarian high-grade serous carcinomas, and identical TP53 mutations have been reported in TICs and in small foci of histologically normal tubal epithelium that diffusely expresses p53, which has been termed “p53 signature.” It has been suggested that p53 signatures are precursors of TICs which in turn precede the development of high grade serous carcinoma. Moreover, it has been proposed that when there is a synchronous TIC and ovarian high-grade serous carcinoma, the fallopian tube is the primary site of origin for the “ovarian” tumor [61].

### 2.4. BRCAness

The term “BRCAness” has been used to describe the phenotypic characteristics that some sporadic OCs share with tumors found in the setting of BRCA germline mutations. The term also reflects that this common biologic behavior comes from molecular defects in the cellular machinery similar to those caused by BRCA mutation [12, 65]. The notion began to form in 1996 after studies of BRCA1/2 genes in sporadic OC showed multiple defects in the BRCA1/2 pathway that would explain a BRCA-like phenotype. BRCA1 and BRCA2 germline mutations are the fundamental defect in hereditary OC where the normal allele of the carrier is inactivated in cancer cells [66, 67]. In sporadic forms, BRCA1/2 somatic mutations are not very common [68] but still are a significant causative gene defect, as shown in extensive genomic analyses of ovarian carcinoma by the Cancer Genome Atlas Research Network [69]. Either genetic or somatic mutations of BRCA1 and BRCA2 are found in approximately 20% of all ovarian tumors [70]; when considering all kinds of BRCA1/2 alterations, including mutations, these have been reported in up to 82% of ovarian tumors [66]. In the absence of BRCA mutations, the BRCAness pattern of biological and clinical behavior seems to be the result of different epigenetic processes. Indeed, epigenetic mechanisms of transcriptional silencing are known to inactivate tumor suppressor genes. BRCA1 protein and mRNA levels in ovarian tumors are decreased or absent in as many as 90% of patient cases without evidence of germline BRCA1 mutations or family history of BRCA-associated diseases [71]. Baldwin et al. demonstrated that aberrant methylation in cytosine residues of CpG dinucleotides in the BRCA1 promoter leads to decreased BRCA1 expression in 5%–30% of ovarian tumors, resulting in BRCAness. Moreover, they showed that 12 of 81 (~15%) ovarian tumors in patients without a family history of OC had evidence of BRCA1 promoter methylation. None of the 12 normal ovaries had evidence of methylation. Tumor and genomic DNA from patients with BRCA1 promoter methylation were screened for the three key founder mutations in the Ashkenazi Jewish population: BRCA1 185delAG, 5382insC, and BRCA2 6174delT. None of the tumors that demonstrated BRCA1 promoter methylation had concurrent BRCA1 mutations. Baldwin et al. also performed immunohistochemistry on paired paraffin-embedded tumor tissues. BRCA1 expression was detected in the nuclei of adjacent stromal cells but in none of the 12 tumors, suggesting BRCA1 inactivation through promoter methylation. Only 5 of these 12 methylated samples exhibited LOH at the BRCA1 locus. While discordant findings between these studies may be a product of biases introduced by population sampling, they suggest the possibility of alternative sites of inactivating BRCA1 methylation not detected in either group's assay [72]. A subsequent study indicated that BRCA1 promoter methylation can be an unfavorable prognostic factor compared to either BRCA1 germline mutation or no loss [73]. A more recent report found epigenetic silencing of BRCA1 and BRCA1/2 mutations to be mutually exclusive; patients with epigenetic BRCA1 silencing showed a similar prognosis as noncarriers [69]. Although LOH for the BRCA locus has been noted in sporadic breast cancer [74], the importance of this mechanism has not been verified in OC. BRCAness could also emerge from defects in genes whose function either affects or is affected by normal BRCA gene function.

### 2.5. Treatment of BRCA-Associated Ovarian Cancer

Despite being more aggressive than sporadic ovarian carcinomas, those arising in BRCA mutation carriers show higher susceptibility to platinum-salts and other DNA-damaging agents. Platinum-salts interfere with DNA cross-links creating double-strand breaks in the DNA helix, which cannot be repaired in BRCA due to HR deficiency. Studies demonstrated an improved long term-survival in women with OC treated with platinum-salts if compared to sporadic OC [75]. Intraperitoneal cisplatin chemotherapy has been shown to lead to favorable long term outcome in advanced OC women with BRCA mutation [76]. Similar outcomes were seen using pegylated liposomal doxorubicin [77–79].

In the last years the inhibition of the poly(ADP-ribose) polymerase enzyme (PARP) has emerged as a promising therapeutic approach in BRCA-associated OCs. PARP is a class of proteins which produces large branched chains of poly(ADP) ribose (PAR) from NAD+. They are involved in a number of cellular pathways including transcriptional regulation, DNA replication, and DNA damage repair [4, 80]. Of the numerous PARP protein detected, PARP-1 and PARP-2 were found to be mostly involved in DNA stability [81]. PARP-1 is a highly conserved nuclear enzyme whose main task is to assist the repair of single strand breaks (SSB) through the BER pathway therefore contributing to the maintenance of genomic integrity [82]. Inhibition of PARP generates DNA lesions caused by lack of an efficient repair of SSB lesions that may cause DSBs or collapsed replication forks. This damage requires functional BRCA1 and BRCA2 for DNA repair [3]. In the presence of a BRCA1 or BRCA2 defective background, HR is impaired and therefore PARP inhibition may results in the generation of replication associated DNA lesions that cannot be effectively repaired, leading to decreased chromosomal stability, cell cycle arrest, and/or cell death [81]. It was shown that cell lines lacking wild-type BRCA1 or BRCA2 and the tumors that they form are 1000-fold more sensitive to PARP inhibitors compared to heterozygous mutant or wild-type cells [81, 83]. Wild-type and BRCA1 or BRCA2 heterozygous cells are able to repair DSBs maintaining cell viability. Patients with BRCA-associated cancers usually lack wild-type BRCA1 or BRCA2 in tumor cells but normal cells retain a single wild-type copy of the gene. PARP inhibitors are, therefore, highly selectively lethal to cells that lack functional BRCA1 or BRCA2 and are associated with minimal toxicity to normal cells [4].

The oral PARP inhibitor olaparib has shown to be well tolerated in phase I studies. A phase II study recruiting patients with BRCA1/2 mutations with recurrent OC demonstrated the efficacy of single agent olaparib with a median duration of response of 9.5 months and with a 66% of clinical benefit rate with the dose of 400 mg twice a day [84]. At ASCO 2013 annual meeting Ledermann et al. [85] presented the results of a preplanned subgroup analysis of maintenance therapy with olaparib in platinum-sensitive relapsed serous OC and BRCA mutation from a previous randomized phase II study [86]. Olaparib maintenance treatment led to the greatest clinical benefit in patients with BRCA mutation. Besides olaparib other promising PARP inhibitor agents being investigated are niraparib, veliparib, and rucaparib. Preclinical data have shown a degree of synergy between PARP inhibitors and chemotherapy; however a recent randomized trial of olaparib in association with paclitaxel and carboplatin failed to show substantial benefit [87]. Future challenges in BRCA mutated OC will include the overcoming of PARP inhibitors resistance that ultimately develops in all patients and the identification of biomarker other than BRCA to select patients with homologous recombination defect that will benefit the most from targeted treatment.

### 2.6. Prognosis

Evidence exists that OC patients carrying germline BRCA mutations have an improved prognosis in comparison to sporadic cases. A pooled analysis of 26 observational studies was recently carried out to explore survival differences between BRCA mutation carriers (1213 overall) and noncarriers (2666); BRCA mutation carriers showed a more favorable prognosis than noncarriers [88].

A favourable prognosis for BRCA mutation carriers with OC was also supported by a recent retrospective study on 190 ethnically heterogeneous patients, 90 of whom were BRCA mutation carriers. A significantly longer overall survival was reported in mutation carriers compared with noncarriers (median overall survival: 93.6 months versus 66.6), although no difference was observed in progression-free survival [89]. Improved rates of progression-free and overall survival in BRCA1/2 carriers were also found in an Australian series of OC patients tested for BRCA regardless of family history [47].

By comparing OC outcome in BRCA1 versus BRCA2 mutation carriers, Liu et al. [90] failed to find any difference in event-free survival, while a nonsignificant increase in 5-year overall survival was observed in BRCA2 (75%) in comparison to BRCA1 carriers (61%).

An Italian retrospective study found a survival advantage in BRCA2-positive versus BRCA1-positive OC patients, the median progression free survival being 45.46 months in the former and 27.2 in the latter [91]. The better prognosis of BRCA2 mutation carriers is supported by another retrospective study on 190 patients (47 mutation carriers and 143 noncarriers), where multivariate analysis showed improved overall survival in BRCA2 carriers but not in BRCA1 carriers, in comparison with noncarriers [92].

A study on the outcome of primary surgical cytoreduction in 69 BRCA mutated in comparison to 298 wild-type patients with high-grade serous ovarian carcinoma (FIGO stage IIIC-IV) demonstrated that BRCA status was not associated with residual tumor volume on multivariate analysis, which led the authors to conclude that improved survival in BRCA carriers was not attributable to an increased rate of optimal tumor debulking [93].

Taken together, these studies consistently support a significantly improved survival in BRCA2 mutation carriers in comparison to sporadic OC patients, while for BRCA1 carriers the advantage, if any, seems smaller.

### 2.7. Correlations between BRCA Deficiency and Mitochondrial Function and Potential Implications for Therapy

A recent study demonstrates that BRCA1 mutations in breast cancer drive oxidative stress and glycolytic transformation of the tumor. Loss of BRCA1 function leads to hydrogen peroxide generation in both epithelial breast cancer cells and neighboring stromal fibroblasts and promotes the onset of a reactive glycolytic stroma. Importantly, these metabolic changes can be reversed by antioxidants, which potently induce cancer cell death [94]. Furthermore our group has recently contributed to a study in which a correlation was shown between expression levels of BRCA2 and mitochondrial DNA (mtDNA) in prostate cancer [95]. MtDNA-depleted and HR-deficient cells appear to exhibit more than 50% reduction in BRCA2 protein expression through a posttranslational mechanism. For these reasons mtDNA-depleted cell lines die after PARP inhibition. Taken together these studies suggest a biologic relationship between mitochondria and BRCA genes. Mitochondrial dysfunction and loss of BRCA2 in sporadic cancer lead to nuclear genomic instability, cumulative mutations, and tumor progression but also enhance cancer cells sensitivity to apoptosis induced by PARP inhibitors [95]. Furthermore, by studying prostate tissue specimens from prostate cancer patients a direct correlation between presence of mtDNA large deletions and loss of BRCA2 protein* in vivo* was found, suggesting that mtDNA status might serve as a marker to predict therapeutic efficacy to PARP inhibitors. Further studies are warranted to confirm this correlation.

## 3. Prevention of BRCA-Associated Ovarian Cancer

### 3.1. Identification and Cancer Risk Assessment in BRCA Mutation Carriers

The detection of a mutation in a patient with breast or ovarian cancer allows for identification of asymptomatic mutation carriers in the family. Such predictive genetic testing should be performed in the framework of a comprehensive genetic counseling process, aimed at facilitating informed decisions about testing and risk-reducing options.

Carriers of BRCA mutations are at increased risk of both breast and ovarian cancer; such risks are consistently estimated to be higher in BRCA1 than in BRCA2 mutation carriers; moreover, the risk is higher for breast than for OC. Estimates of cancer risk are variable across different studies; nevertheless, two large meta-analyses, resulting from a total of 31 published studies, estimated an OC risk to age 70 of 49% for BRCA1 and 18% for BRCA2 [96] and of 39% for BRCA1 and 11% for BRCA2 [97]. The risk for breast cancer was 55% to 65% for BRCA1 and 45% to 47% for BRCA2 mutation carriers. However, the penetrance of BRCA mutations is likely to be overestimated in published studies, as carriers are mostly ascertained based on the clustering of cancer in the family, which may reflect the presence of additional risk factors modifying the penetrance.

In Europe and the United States, the onset of familial OC is generally reported to be 5 to 10 years earlier than that of sporadic OC, but the early onset is probably limited to BRCA1 mutation carriers. Indeed, Risch and colleagues reported a mean age at OC onset of 51.2 years for BRCA1 mutation carriers and of 57.5 years for BRCA2 positive women [98]. Similarly, in the series described by Liu et al, the median age at OC diagnosis was significantly younger in BRCA1 (51.1) in comparison to BRCA2 carriers (55.4) [90]. In Japan, age at onset of familial OC is similar to that in sporadic OC, although BRCA2-related OC tends to develop at a later age than sporadic OC [99].

### 3.2. Management of BRCA Mutation Carriers

Options to reduce the risk of OC or fallopian tube cancer in BRCA mutations carriers include surveillance, chemoprevention, and surgery [100]. Nevertheless, screening and prevention guidelines are generally based on nonrandomized trial and observational studies [101]. Although based on expert consensus (lowest level of evidence), the National Comprehensive Cancer Network (NCCN) guidelines provide the most comprehensive guidelines currently available [100].

#### 3.2.1. Surveillance for Ovarian, Fallopian, and Primitive Peritoneal Cancer

Recommended surveillance should be performed with pelvic examination, transvaginal ultrasound, and CA-125 levels every 6 months beginning at age 30 or 5–10 years earlier than the youngest relative diagnosed with OC [100, 102]. Little is known about the mechanism or timing of progression from localized to disseminated ovarian cancer. Early detection of the ovarian cancer is less effective than breast cancer screening and these surveillance methods have not been demonstrated to reduce OC mortality [101]. Three large randomized controlled trials to determine whether screening for OC, compared with no screening, can achieve earlier diagnosis and decreased mortality have been or are being conducted in the United Kingdom, Japan, and the United States. Mortality data from the Prostate, Lung, Colorectal, and Ovarian (PLCO) Cancer Screening Trial in the UK have been reported, showing no change in the stage of cancer detected by screening and no decrease in cancer-specific or overall mortality for women who underwent annual screening (four years of transvaginal ultrasound and six years of CA-125 serum levels) [103–105]. The potential risks associated with screening for OC must also be considered. A positive screening result for OC most often is followed by surgery (either laparoscopy or laparotomy). Invasive procedures are associated with physical and psychological morbidity, a small risk for serious complications, and substantial financial costs. The PLCO trial reported that 15 percent of women who underwent surgery for false positive findings experienced a serious complication related to surgery [103].

In conclusion surveillance for OC in BRCA positive patients with pelvic exam, transvaginal ultrasound, and serum levels of CA-125 is not invasive, but its impact on early detection and mortality is low and may be associated with emotional distress [106].

#### 3.2.2. Risk Reduction Surgical Options

Since surveillance for ovarian, peritoneal, and fallopian tube cancer has not been proven to be effective, prophylactic salpingooophorectomy is actually recommended to BRCA positive women by the age of 40 years and upon completion of child bearing [100].

Salpingooophorectomy in BRCA patients should be performed considering specific issues and adequate surgical details. Specific preoperative counselling and informed consents should be obtained [107]. This surgery reduces the risk of ovarian and fallopian tube cancer by 75–96% and of breast cancer by approximately 50% if performed after natural menopause [108]. The procedure, however, does not eliminate the risk of primary peritoneal cancer, that, after prophylactic surgery, has been estimated to be between 2 and 4% [109]. The procedure begins with a thorough inspection of the peritoneal cavity, comprehensive of pelvic organs, gastrointestinal surfaces, liver, omentum, and pelvic, abdominal, and diaphragmatic peritoneal surfaces. Any suspected lesion should be biopsied and sent for frozen section. Abdominal cavity should be washed with saline and a sample obtained for cytology. This procedure is generally adequately performed by laparoscopy, even in presence of adhesions due to previous abdominal procedures.

Salpingooophorectomy requires complete removal of the ovary and the fallopian tube; the procedure should therefore be preceded by an accurate periadnexal adhesiolysis to obtain clear margins and simple handling of the structures. Peritoneum should be entered laterally to the ovarian vessels and visualization of ureter, ovarian artery, and veins obtained. Ovarian vessels should be coagulated and cut 1-2 cm away from the ovary to obtain clear margins and to verify the absence of vascular and lymphatic involvement. The peritoneum next to the ovary has to be cut with large margins to identify microscopic neoplastic foci. Finally, uteroovarian ligament and fallopian tubes should be coagulated and sectioned as close to the uterine wall as possible [110].

Fallopian tube and ovary are removed intact through an endobag to avoid spillage in the abdominal cavity and sent for frozen section. For logistic or organization issues frozen section could be missed, but the necessity of second surgery has to be discussed with the patient during preoperative counselling [111].

The removal of the uterus is not mandatory when preoperative imaging has excluded endometrial or myometrial disease; the risk of remnant of the intramural fallopian tube is negligible, but it should be discussed with the patient. Hysterectomy simplifies hormone replacement therapy and eliminates the possible increased risk of endometrial cancer and the possible increased risk of serous carcinoma of the uterus [112, 113].

Because of the tubal origin of serous ovarian carcinoma [114], some authors advocate the removal of the fallopian tubes, or fimbriectomy, as first step in prophylactic surgery for BRCA 1 and BRCA 2 patients as a bridge procedure for young patients willing to postpone oophorectomy after the fifth decade of life [115]. The procedure does not imply hormonal deficiency, but the safety and validity of this procedure should be confirmed by a multi-institutional study. Women who undergo radical fimbriectomy should continue to receive regular surveillance.

Finally, single port procedures are now available and this low aesthetic impact surgical access could be proposed to BRCA 1 and BRCA 2 patients [116].

The pathologic examination of specimens from risk-reducing salpingooophorectomy requires special attention. To this aim, the BRCA mutation status should be always shared with the pathologist, since in patients with benign gynecologic disease only one slide from the fallopian tube and ovary is normally reviewed, while in the setting of a known genetic predisposition to OC, most pathologists will submit the entirety of the fallopian tubes and ovaries for microscopic examination. The SEE-FIM (sectioning and extensively examining the fimbriated ends) protocol is widely used. Basically, this involves serially sectioning the tube meticulously, stopping before the fimbriae. The fimbria is amputated and sectioned longitudinally, thereby maximizing exposure. Deeper sections need to be obtained if foci of atypia are to be identified histologically. Foci of in situ or invasive occult carcinoma may be very subtle and are often less than 1 mm in maximum diameter. Microscopic occult carcinomas have been identified in these specimens in about 2% to 11% of BRCA mutation carriers, generally involving the tubal fimbriae [117–119].

## 4. Conclusions

Literature data demonstrates that OC arising in BRCA1/2 mutation carriers have peculiar molecular, pathological, and clinical features. Since a nonnegligible proportion of newly diagnosed OC patients are expected to carry such mutations, BRCA1/2 mutational analysis would help, in the future, to tailor OC management according to BRCA status. In addition, this would allow the subsequent identification of asymptomatic carriers who would benefit from targeted interventions for high-risk women.

## Figures and Tables

**Table 1:** Distribution of morphology and grade of ovarian tumors arising in BRCA1 and BRCA2 mutation carriers from the Consortium of Investigators of Modifiers of BRCA1/2 (CIMBA) [60].

Factor		BRCA1 *n* (%)	BRCA2 *n* (%)	Total *n* (%)
Morphology	Serous	534 (66)	191 (70)	725 (67)
	Mucinous	11 (1)	4 (1)	15 (1)
	Endometrioid	94 (12)	33 (12)	127 (12)
	Clear cell	8 (1)	8 (3)	16 (1)
	Other	166 (20)	36 (13)	202 (19)
	Total	**813**	**272**	**1,085**

Grade	1	17 (3)	11 (6)	28 (4)
	2	104 (20)	37 (21)	141 (20)
	3	407 (77)	128 (73)	535 (76)
	Total	**528**	**176**	**704**
